# Intermittent thoracic resuscitative endovascular balloon occlusion of the aorta improves renal function compared to 60 min continuous application after porcine class III hemorrhage

**DOI:** 10.1007/s00068-022-02189-2

**Published:** 2022-12-05

**Authors:** Andreas Brännström, Michael Hultström, Jenny Gustavsson, Zabih Aurfan, Mattias Günther

**Affiliations:** 1 Department of Clinical Science and Education, Södersjukhuset Karolinska Institutet, Sjukhusbacken 10, S1, 11883 Stockholm, Sweden; 2 Department of Medical Cell Biology, Uppsala University, Uppsala, Sweden; 3 Department of Neuroscience, Karolinska Institutet, Stockholm, Sweden

**Keywords:** iREBOA, cREBOA, Hemorrhage, Trauma, Acute kidney injury

## Abstract

**Background:**

Resuscitative Endovascular Balloon Occlusion of the Aorta (REBOA) may be considered for stabilization of patients with hemorrhage from below the diaphragm. Occluding the aorta is a powerful means of hemorrhagic control but is also associated with acute kidney injury, which increases mortality in trauma patients. Allowing for intermittent distal blood flow during REBOA application (iREBOA) could decrease this risk, but circulatory consequences have not been sufficiently elucidated. Therefore, we investigated circulatory effects and the renal artery blood flow (RBF) in iREBOA versus continuous, complete aortic occlusion (cREBOA).

**Methods:**

In a porcine model of uncontrolled class III hemorrhage (34% estimated total blood volume, mean 1360 mL), swine (*n* = 12, mean weight 60.3 kg) were randomly assigned to iREBOA: 3-min full deflation every 10 min (*n* = 6), or cREBOA (*n* = 6), for 60 min of thoracic (zone I) application. The animals then underwent 60 min of reperfusion (critical care phase).

**Results:**

Survival was 100% in iREBOA and 83% in cREBOA. The intermittent balloon deflation protocol was hemodynamically tolerable in 63% of reperfusion intervals. Systolic blood pressure decreased during the reperfusion intervals in iREBOA animals (mean 108 mm Hg versus 169 mm Hg; *p* < 0.005). No differences were detected in heart rate, cardiac output or stroke volume between methods. Troponin I increased in cREBOA after 60 min (mean 666–187 ng/L, *p* < 0.05). The norepinephrine requirement increased in cREBOA during reperfusion (mean infusion time 12.5–5.5 min; *p* < 0.05). Total ischemic time decreased in iREBOA (60.0–48.6 min; *p* < 0.001). RBF increased in iREBOA during balloon deflations and after 60 min reperfusion (61%–39% of baseline RBF; *p* < 0.05). Urine output increased in iREBOA (mean 135–17 mL; *p* < 0.001). Nephronal osteopontin, a marker of ischemic injury, increased in cREBOA (*p* < 0.05).

**Conclusion:**

iREBOA was survivable, did not cause rebleeding, decreased the total ischemic time and increased the renal blood flow, urine output and decreased renal ischemic injury compared to cREBOA. Intermittent reperfusions during REBOA may be preferred to be continuous, complete occlusion in prolonged application to improve renal function.

**Supplementary Information:**

The online version contains supplementary material available at 10.1007/s00068-022-02189-2.

## Background

Resuscitative Endovascular Balloon Occlusion of the Aorta (REBOA) may be considered for stabilization of trauma patients with hemorrhage from below the diaphragm who are not responding to standard resuscitation [1]. The balloon device is positioned in the thoracic, descending aorta (zone 1) or below the renal arteries in the abdominal aorta (zone 3) depending on the anatomic location of vascular injury. The minimally invasive procedure may replace open thoracic aortic cross-clamping after non-compressible torso hemorrhage, a situation associated with high mortality [2–4]. Research during the past decade of global physiological effects of REBOA has shown that acute kidney injury (AKI) constitutes an independent risk factor for mortality and prolonged intensive care after trauma and may occur as a result of REBOA [5–10]. Furthermore, myocardial injury occurs during prolonged complete thoracic REBOA [11]. Therefore, current recommendations state a maximum of 30 min, zone 1 REBOA intervention [1]. Organ damage and ischemic injury cause multiple organ dysfunction, a major cause of mortality > 24 h after trauma [12]. It is possible that permissive distal organ perfusion during REBOA reduces ischemic injury. REBOA with smaller devices and partial or intermittent distal aortic flow led to fewer complications in animal models and humans [13]. Today, there are two principal methods of achieving distal aortic flow: partial and intermittent REBOA. Partial REBOA is associated with improved mesenteric perfusion, decreased acidosis, decreased myocardial load [14, 15] and decreased ischemic organ injury compared to intermittent REBOA [16]. Intermittent REBOA is associated with prolonged survival and decreased acidosis compared to continuous REBOA [17]. However, both methods have limitations. Partial REBOA is technically challenging and may not be feasible in situations with limited capabilities such as in a prehospital environment or in combat operations. Intermittent REBOA may cause rebleeding and hemodynamic instability. The relation between intermittent REBOA and distal organ perfusion, and the amount of reperfusion required for improved renal function have not been sufficiently elucidated. The regulation of organ blood flow is complex and multifactorial in REBOA applied after trauma and hemorrhagic shock, and the reduction in renal blood flow may continue after REBOA release [18]. Therefore, we investigated circulatory effects, renal artery blood flow and markers for renal injury, Osteopontine, NGAL and Vimentin, for 60 min of intermittent (iREBOA) versus continuous, complete (cREBOA) zone 1 REBOA, in a model of potentially lethal and uncontrolled hemorrhage. We hypothesized that iREBOA would improve the blood flow in the renal arteries and decrease markers of renal injury.

## Methods

**Fig. 1:**
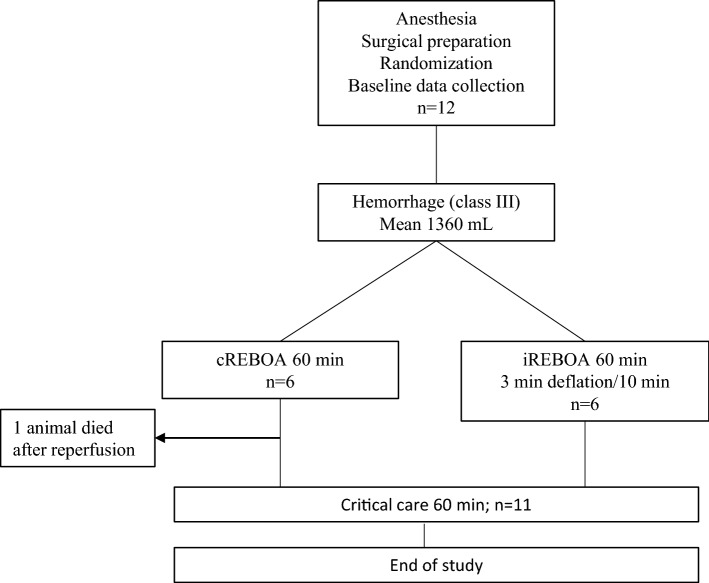
Experimental design

This study was approved and conducted in accordance with the Swedish regional ethics approval board for animal research (ethical approval ID S3-15 and 1470). Castrated crossbred male swine, (*n* = 12, mean weight 60.3 kg), were utilized in a randomized (closed envelope), prospective experimental study design including five phases (Fig. 1). Animals were assigned to continuous, complete 60-min zone 1 aortic occlusion (cREBOA; *n* = 6) or a 3-min full deflation interval every 10 min for 60 min (iREBOA; *n* = 6) followed by 60-min reperfusion after REBOA intervention.

### Anesthesia

The animals were sedated with 150 mg tiletamine/zolazepam (Zoletil 100 Vet) and 6 mg medetomidine (Domitor) before transport to the laboratory facility. After recording of weight, the animals were placed supine on a standard operating table, pre-oxygenated with 100% O_2_ from a nose cone and the left auricular vein was cannulated. Non-invasive blood pressure monitoring (NIBP) was placed on the hind leg and oxygen saturation was monitored from a tail probe. Three intracutaneous EKG electrodes were placed. Anesthesia was induced with pentobarbital 6 mg/kg, atropine 0.02 mg/kg and 2.5 µg/kg fentanyl. Oral intubation with an 8F endotracheal tube was performed and anesthesia was further maintained with continuous infusion of ketamine 25 mg/kg/h (Ketaminol Vet 100 mg/ml), midazolam 0.0485 mg/kg/h (Midazolam Hameln 1 mg/ml) and fentanyl 3.5 µg/kg/h (Fentanyl B. Braun 50 µg/ml). Mechanical ventilation (Hamilton C2 ventilator, Hamilton medical, Geneva Switzerland) was adjusted to maintain tidal volumes of 7 ml/kg. Ventilator settings were as follows: respiratory rate 20/min during REBOA and adjusted to 25/min after balloon deflation; FiO_2_ 21%; peak inspiratory pressure 13–15 cm H_2_O and peak expiratory end pressure 5 cm H_2_O. A bolus of 500 ml Ringers-acetate was given to adjust variances in baseline fluid balance and subsequently throughout the experiment with 3 ml/kg/h to compensate for insensible losses.

### Surgical preparation and cannulation

The right external jugular vein was surgically exposed and a 7.5F Swan-Ganz pulmonary artery catheter (Edward Lifescience) was positioned for collection of core body temperature, central venous pressure, cardiac output and mixed venous oxygen saturation. An arterial catheter was positioned in a brachial artery with ultrasound guidance and used for pressure and blood sampling. The left kidney was exposed via midline laparotomy, and a fitted perivascular ultrasound flow probe (Transonic Systems Inc., NY, USA) was positioned around the renal artery. A 1.5 mm cannula was positioned in the renal vein for blood sampling. A suprapubic urine catheter was placed before the midline laparotomy incision was temporarily closed with staples and connected to an electronic weight scale for continuous monitoring. The common femoral artery was surgically exposed for later hemorrhage. The contralateral femoral artery was cannulated with a 7F introducer sheath (Merit Medical Systems Inc., UT, USA) via ultrasound guided, seldinger technique. A 6F, 15 mm diameter/50 cm shaft REBOA catheter (REBOA Medical, Båstad, Norway) was advanced through the introducer to a supra-celiac position-based topical measurement in a standardized procedure and maintained in position. Complete baseline data were then collected.

### Hemorrhage shock and resuscitation

A hybrid hemorrhage protocol of 34% estimated total blood volume (67 ml/kg) was utilized to produce a class III hemorrhage with severe hypotension. Hemorrhage was initiated by blood withdrawal through a 7F femoral sheath to citrate bags (2 × 450 mL) followed by transection of the femoral artery and collection of extravasated blood to a container placed on an electronic weight scale. Blood spill was collected in gauzes and weighted. The free bleeding was periodically paused by manual pressure of the femoral artery, while total blood loss was calculated and temporarily clamped during a period of 15 min. The femoral artery was unclamped at the onset of the intervention phase, and for the remainder of the experiments, in both study groups to evaluate rebleeding during balloon deflation intervals. The hemorrhage protocol was 100% lethal without resuscitation in pilot studies (data not shown). After completion of the post-hemorrhage data collection, one bag of whole blood (450 mL) was administered in combination with simultaneous inflation of the prepositioned REBOA balloon (8 mL NaCl). If systolic blood pressure decreased < 80 mm Hg during reperfusion intervals in the iREBOA group, a rescue occlusion (full inflation of the balloon) was conducted. Hemostasis was confirmed by visual inspection. Animals assigned to cREBOA had full inflation of the balloon, and animals assigned to iREBOA had a 3-min full deflation interval every 10 min. At 60 min, the transected femoral artery was clamped using standard hemostatic forceps. The animals were transfused with one bag of whole blood (450 mL) immediately prior to final deflation of the balloon. Except for the transfused blood, no other resuscitation fluids were administered during the induction of hemorrhage shock or REBOA intervention. After the 60-min intervention phase, the animals were monitored for an additional 60-min reperfusion phase during which the animals were given a rapid infusion of 2000 mL crystalloids (Ringer´s Acetate). Norepinephrine infusion was started (0.1 µg/kg/min) to maintain a systolic blood pressure > 80 mm Hg. Norepinephrine was paused when systolic blood pressure reached 100 mmHg. Criteria of death were asystole on EKG in combination with etCO_2_ < 2 kPa and MAP < 20 mmHg.

### Data collection and analyses

Hemodynamic parameters and EKG were continuously recorded. Total ischemic time was calculated for 60 min—total time between each deflation and re-inflation. Re-inflation of the balloon for each animal and data are presented as means. Arterial and renal vein blood chemistry were analyzed at baseline, hemorrhage completion, 5, 10 and every 10 min after REBOA inflation. Norepinephrine infusion was automatically recorded. Urine output was continuously recorded by an electronic weight scale. Troponin I was analyzed at baseline and post intervention (60-min REBOA) and reperfusion phases. Creatinine (8L24, IDMS traceable enzymatic reagent, Abbott Laboratories, Abbott Park, IL, US) was analyzed on a BS380 instrument (Mindray, Shenzhen, China). The total coefficient of variation (CV) for the creatinine method was 1.5% at 87 μmol/L.

### Immunohistochemistry

For injury marker detection, 10 µm slides from each swine in cREBOA and iREBOA groups were cut from the cortex of fresh frozen kidneys (stored in − 70 °C). Primary antibodies were diluted in 5% donkey serum and 95% primary buffer (0.01 M PBS + 0.1% NaN3 + 0.3% triton + 5% bovine serum albumin). NKCC2 antibody (1:2000, Everest Biotech, EB09143) was combined with either Osteopontin (1:400 Abcam 8448, Product ID: GR5225573-4), NGAL (1:1000, BioSite, Product ID: GTX60965) or Vimentin (1:100, BioGenex, Product ID: MU074-UC). Slides were rinsed for 10 min in PBS and incubated with primary antibodies overnight at 4 °C. Then, slides were rinsed and incubated with secondary antibodies for 1 h in room temperature, and all dilutes were 1:400 in 5% swine serum and secondary buffer (0.01 M PBS + 0.1% NaN3 + 0.3% triton). A mixture of Cy2 (Jackson ImmunoResearch, Product ID: 705225147) and of Cy3 (Jackson ImmunoResearch, Product ID: 715165151) was used for slides stained with Osteopontin or Vimentin. For slides stained with NGAL, a mixture of Cy2 (Jackson ImmunoResearch, Product ID: 705225147) and of Cy3 (Jackson ImmunoResearch, Product ID: 711165152) was used. Slides were then rinsed in 0.01 M PBS and mounted with Mowiol.

### Image analysis

X20 magnified high-power-fields (HPF) were acquired using a Nikon Eclipse Ni-E microscope equipped with an AndorZyla VSC-02613 camera. Three HPFs per slide, three slides per subject, were acquired for each injury marker. Totally 27 HPFs, nine for each injury marker, were analyzed for each swine. The integrated intensity (staining intensity × area) in the HPF of each injury marker was analyzed using specified macros in ImageJ (Supplemental Table 1). The integrated intensity was measured where each injury marker and NKCC2 were co-localized.

### Statistical analyses

All statistical calculations were made using GraphPad prism v8.1.1 (GraphPad Software Inc., La Jolla, Ca, USA). *P* ≤ 0.05 was considered statistically significant. Comparisons of hemodynamic variables, urine output and metabolic changes were performed using repeated measures, two-way analysis of variance (ANOVA). Sidak’s multiple comparisons test was applied for post hoc corrections. For hemorrhage volume, norepinephrine infusion time, total ischemic time and organ specific blood markers, unpaired *t* tests were used. For immunohistochemical stainings, unpaired, two-tailed *t* test was used. Primary outcome was renal blood flow. Secondary outcomes were total ischemic time, urine output, creatinine, troponin I, norepinephrine and metabolic parameters.

## Results

**Fig. 2:**
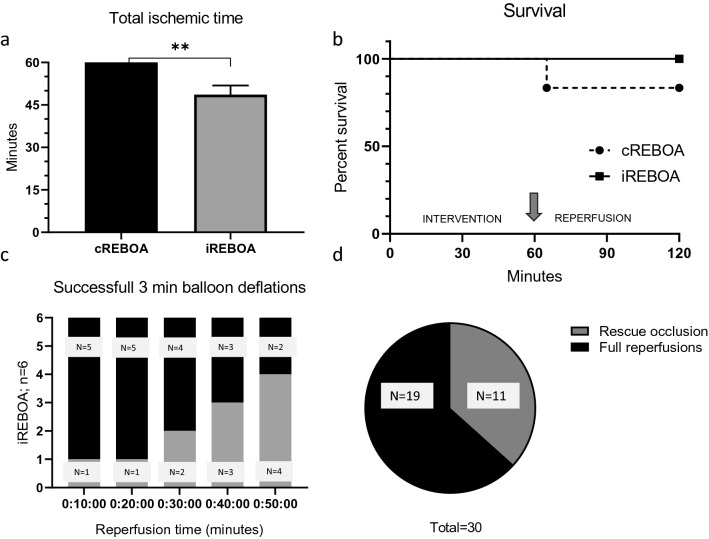
Total ischemic time, survival and successful intermittent reperfusions. **a** Difference in total ischemic time between continuous occlusion and the iREBOA protocol (48.6 min ± 1.3 min versus 60 min ± 0 min. **b** One animal died after balloon deflation and continuous 60 min thoracic REBOA. **c** Ratio of successful 3 min balloon deflations at each 10 min interval (range: 10 min = 83%; 50 min = 33%). **d** Ratio of successful 3 min balloon deflations; 63% of total. **p < 0.01

**Fig. 3:**
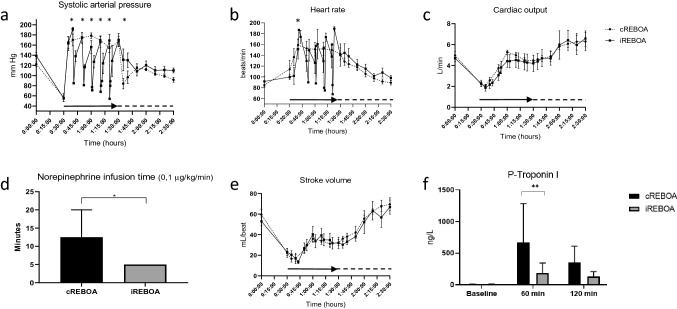
Circulatory consequences of iREBOA versus cREBOA. **a** Systolic arterial pressures in iREBOA and cREBOA intervention and reperfusion. **b** Heart rates decreased after balloon deflation during thoracic REBOA (*p* < 0.05). **c** Cardiac output did not differ between groups. **d** Vasopressor requirements after balloon deflation. Reperfusion increased vasopressor requirements in cREBOA. **e** Stroke volume did not differ between groups. **f** Release of Troponin I increased after 60 min cREBOA (*p* < 0.05). Black arrow and horizontal dotted line represent REBOA intervention time and reperfusion time, respectively

Baseline parameters did not differ between groups (data not shown). One REBOA balloon malfunctioned during first inflation and was quickly replaced. A zone 1 location of the REBOA balloons was confirmed in all animals during post-mortem laparotomy. Average blood loss was 1360 mL (cREBOA: 1382 mL; iREBOA: 1337 mL). No significant rebleeding from the femoral artery was observed during reperfusion intervals in the iREBOA animals. Total ischemic time was lower in the iREBOA group (48.6 min ± 1.3 min versus 60 min ± 0 min in cREBOA) (*p* ≤ 0.001) (Fig. 2a). Survival was 100% (iREBOA; *n* = 6 of 6) and 83% (cREBOA; *n* = 5 of 6) (Fig. 2b). The death occurred within 5 min of the reperfusion phase, after total balloon deflation. Ventricular fibrillation was observed in EKG before the criterion of death was reached and no additional resuscitation was performed. Available data were included, and the animal was excluded in further analyses for the remainder of the study. The ratios of successful three minutes reperfusion intervals are shown in Fig. 2c and d. The need for rescue occlusion due to severe hypotension (Figs. 2c, 3a) showed a linear increase during the 60 min intervention. A full 3-min reperfusion was successfully conducted in 63% of total, ranging from 83 to 33% between 10- and 50-min intervention time. Individual values of reperfusion times are presented in Table 1.

**Table 1:** Individual reperfusion times in iREBOA

Time	Individual reperfusion times iREBOA (s)				
Animal	T_10_	T_20_	T_30_	T_40_	T_50_
1	180	120	67	48	54
2	180	180	180	105	63
3	57	180	180	180	63
4	180	180	180	180	180
5	180	180	60	27	15
6	180	180	180	180	180
Mean	160	170	141	120	93

Note that, rescue occlusion occurred if SBP < 80 mm Hg. Rescue-occlusions occurred unpredictably and it was not possible to anticipate a hemodynamically vulnerable situation following balloon deflation during the protocol

Systolic blood pressure increased from a mean 56 mm Hg to 164 mm Hg (198%) in both groups with balloon inflation (cREBOA: mean 57 ± 19 mm Hg to 163 ± 34 mm Hg; iREBOA: mean 55 ± 16 mm Hg to 165 ± 29 mm Hg). SBP decreased in the iREBOA group during balloon deflations (Fig. 3a) with a total of 11 hypotensive events (Fig. 2d). Five minutes after final balloon deflation, the cREBOA animals had lower blood pressure (Fig. 3a). Cardiac performance showed no significant differences, however, there was a clear trend toward lower heart rate in the iREBOA group during balloon deflations with no difference in cardiac output (Fig. 3b, c, e). Norepinephrine infusion time increased after reperfusion and continuous occlusion (cREBOA: mean 12.5 min; iREBOA: mean 5 min; *p* < 0.05) (Fig. 3d). Troponin I increased in cREBOA animals (mean 666 ng/L) after 60 min intervention compared to iREBOA (mean 187 ng/L). Troponin increased during REBOA intervention and showed a similar trend toward normalization after 60 min reperfusion (cREBOA: 352 ng/L; iREBOA: 127 ng/L) in both groups (Fig. 3f).

**Fig. 4:**
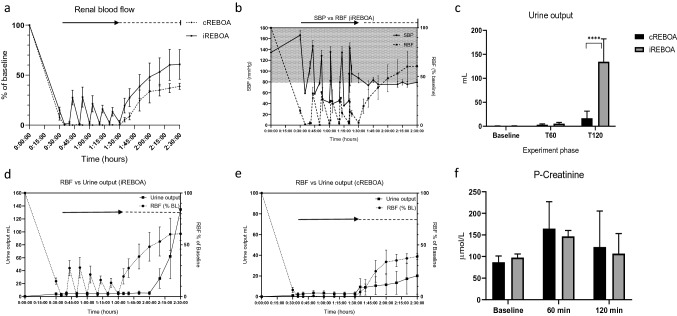
Renal consequences of iREBOA versus cREBOA. **a** Return of renal blood flow (RBF) increased during intermittent reperfusions and in iREBOA animals after 60 min reperfusion (mean difference = 11.79%; *p* < 0.05). **b** The association between proximal systolic blood pressure (SBP) and RBF (white area = hypotension; threshold for balloon rescue occlusion 80 mm Hg). **c** Urine output reperfusion increased in iREBOA after 60 min. **d**, **e** The association between RBF and urine output. **f** Plasma creatinine levels did not differ. Black arrow and horizontal dotted line represent REBOA intervention time and reperfusion time, respectively

Renal blood flow increased during reperfusion intervals in iREBOA animals and after 60 min reperfusion (Fig. 4a). The return of blood flow in the renal artery was 27.5% of baseline values during the first 3 min reperfusion where after it decreased gradually to 13.5%. Peak return of RBF after reperfusion was 61% (iREBOA) and 39% (cREBOA), respectively (Fig. 4a). The increase in renal artery blood flow during reperfusion corresponded to a decrease in proximal systolic blood pressure and increased blood flow distal to the balloon (Fig. 4b). Both groups showed a restoration of urine output after 60 min reperfusion, however, the cumulative urine output increased after iREBOA (mean difference 118 mL (± 17 mL; SE 30.38 mL); *p* < 0.005) (Fig. 4c–e). Mean plasma levels of creatinine did not differ between groups at 60 min intervention or after reperfusion. In both groups, creatinine levels increased after 60 min REBOA compared to baseline (Fig. 4f). Creatinine increased 90% (cREBOA; *p* < 0.05) and 51% (iREBOA; p ≤ 0.001) between baseline and 60-min c/iREBOA, respectively (Fig. 4f). During reperfusion, plasma creatinine clearance was 27% in both groups (cREBOA:43 µmol/L; iREBOA 40 µmol/L).

**Fig. 5:**
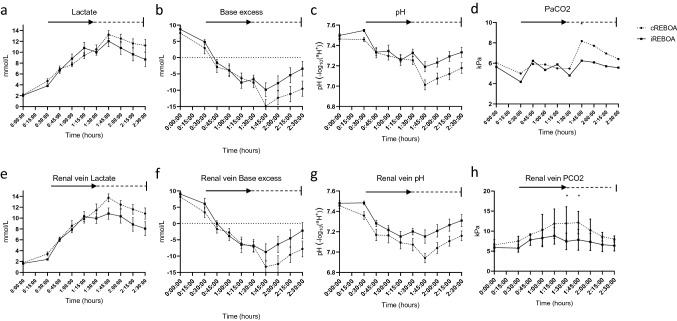
Metabolic consequences of iREBOA versus cREBOA. There were no significant differences. However, there was a trend toward a more severe ischemic insult following 60 min continuous occlusion. pCO2 decreased in both systemic blood and renal vein blood samples immediately following final balloon deflation, indicating a more severe metabolic insult after cREBOA. Black arrow and horizontal dotted line represent REBOA intervention time and reperfusion time, respectively

There was a trend toward increased metabolic acidosis in the cREBOA group during reperfusion phase, however, the mean levels of lactate, base excess or pH did not reach statistical significance (Fig. 5a–c). During reperfusion, cREBOA animals were administered higher norepinephrine doses (cREBOA: mean 75 µg; iREBOA: mean 30 µg; *p* < 0.05) to remain normotensive (Fig. 3d). Crystalloid and autologous whole blood resuscitation did not differ between groups according to the study protocol. PaO_2_ and renal vein PO_2_ showed no differences between groups during the experiments. There was a trend toward increased PaCO_2_ during reperfusion phase in the cREBOA group, and renal vein PCO_2_ increased after cREBOA during first 30 min after balloon deflation (Fig. 5d, h).

**Fig. 6:**
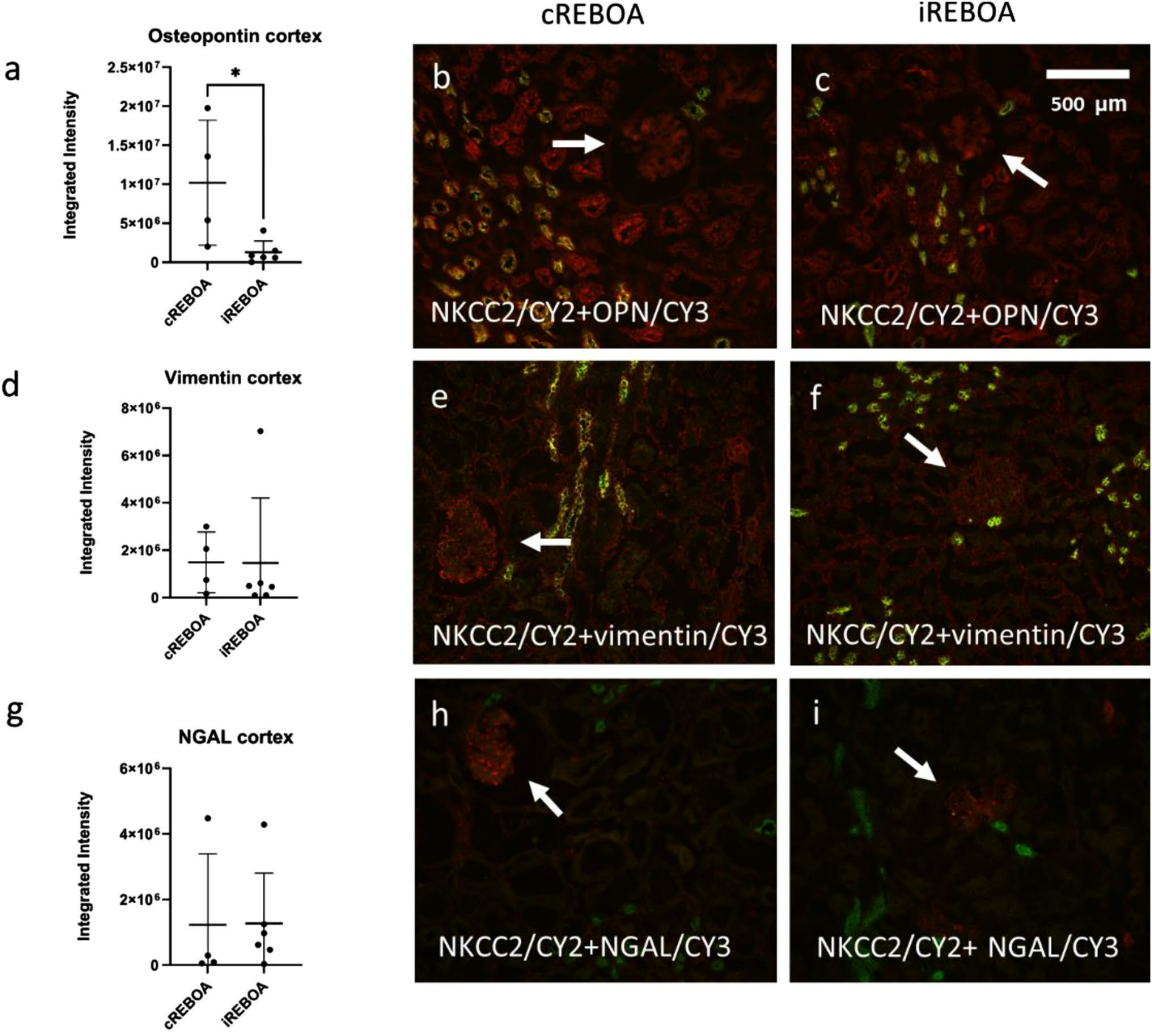
Markers of kidney injury (CY3, red) in the cortex. Double staining with NKCC2 (CY2, green), showing expression of markers in the thick ascending limb of the loop of Henle. **a**–**c** Osteopontin, a marker of ischemic injury, increased in cREBOA vs iREBOA (*p* < 0.05). **d**–**f** Vimentin did not differ between groups. **g**–**i** NGAL did not differ between groups. Arrows mark glomeruli

The integrated intensity (arbitrary units) of Osteopontin, in NKCC2-positive cells (the thick ascending loop of Henle), was significantly higher (*p* = 0.0256) in cREBOA (10184932 ± 8018146) vs iREBOA (1275219 ± 1450814). No significant difference (*p* = 0.9875) in levels of vimentin between cREBOA (1488056 ± 1277875) and iREBOA (1464134 ± 2730189) was detected. Neither was there any difference (*p* = 0.9709) in levels of NGAL in cREBOA (1226757 ± 2168597) compared with iREBOA (1270533 ± 1535600) (Fig. 6a–i).

## Discussion

In this study, we demonstrate that the iREBOA protocol was survivable, did not cause rebleeding, decreased the total ischemic time and increased the renal blood flow and urine output compared to cREBOA.

The rationale for the iREBOA protocol was that an intermittent reperfusion strategy may restore oxygen levels and decrease the anaerobic metabolism before severe ischemic injury could occur, and the rationale for the 60 min application time was to investigate the physiological effects of the reperfusion intervals in relation to a possible extended safe time of supra-celiac REBOA by intermittent balloon deflations. During the development of the protocol, one third of the animals died on prompt deflation of the REBOA balloon after 60 min continuous occlusion, which suggested that deflation of cREBOA may lead to severe circulatory consequences. While this study was not powered to determine survival primarily, our results suggest that while both methods may cause circulatory adverse effects on removal, iREBOA had a lower requirement for norepinephrine and may therefore lead to lower risks on removal. Nevertheless, zone 1 REBOA balloon deflation, even after a short period of total occlusion, should be performed with hemodynamic monitoring and guided by a blood pressure threshold. Until a robust strategy to mitigate the combined effects of hypotension and ischemic reperfusion with associated acidosis and electrolyte derangements has been established [6], the time limit of 30 min continuous (total) zone 1 REBOA should not be exceeded.

Conflicting data have been reported regarding the benefits and risks of intermittent balloon deflation during REBOA [16, 17, 19, 20]. A strict time-based protocol for intermittent reperfusion during REBOA may be easy to manage but risks severe hypotension and should be utilized together with hemodynamic monitoring and guided by blood pressure limits. In this study, the balloon was promptly deflated and re-inflated at each reperfusion interval. It is possible that this approach led to more severe hemodynamic changes than a more stepwise and gradual return of aortic flow. Such techniques have been described and utilized in partial REBOA. However, the return of aortic blood flow during graded deflation of the balloon may be unpredictable and not linearly corresponding to the balloon volume. Moreover, the volume is not transferrable between individuals [21]. Therefore, our technique of prompt balloon deflation allowed for a uniform effect in the animals. The ratio of rescue occlusions was 37%, which suggested that patients should be monitored with invasive arterial blood pressure and that deflation of the balloon should be performed in combination with resuscitation capabilities including volume infusion and vasopressors, even after short intervention times. The ratio of rescue occlusions also suggested that a pressure-based intermittent reperfusion protocol may be superior to a strictly time-based protocol to prevent severe hypotension [16, 17]. We detected a trend toward shorter tolerance of reperfusion times during the 60-min intervention. This may be ameliorated by adjunctive fluid resuscitation. The optimal volume of resuscitation during iREBOA may be guided by hemodynamic parameters and blood gases and should be the focus of future investigations.

Management of REBOA without standard intra-hospital monitoring of the patient’s physiology requires knowledge of the associated organ damage [22]. The kidneys play a vital role in the response to hemorrhage shock. First, the reduced blood pressure secondary to hypovolemia causes decreased glomerular filtration and decreased urine output. Second, the combined effects of decreased renal perfusion and neuro-hormonal activation cause the kidneys to release renin, which stimulates angiotensin II production and causes vasoconstriction to increase blood pressure. In addition, it increases renal reabsorption but this effect may not be sufficient to increase systemic blood pressure in the acute setting, but may increase renal oxygen consumption and cause further hypoxia [23, 24]. The return of renal blood flow during reperfusion intervals was lower than expected. It is likely that the decrease in proximal blood pressure after balloon deflation was caused by a sharp return of blood flow in the abdominal aorta and a significant reduction in cardiac afterload. The renal flow only restored to 31% of baseline. Hence, the renal blood flow could not be predicted by the systemic blood pressure. The mechanism behind the decreased return of renal artery blood flow is likely multifactorial and may be related to increased circulating volume area secondary to redistribution of blood flow in vascular beds in the abdomen and lower extremities. Also, hyperemia after ischemia may contribute to the decreased RBF [25]. The restoration of renal blood flow after the 60-min reperfusion phase increased in iREBOA but only reached 61% of baseline. The mechanism may be related to renal vascular constriction due to endothelial hypoxia and possibly also to central regulation due to baroreceptor signaling from the sharp decrease in blood pressure following balloon deflation, which should be the focus of future investigations. The regulation of renal blood flow is complex and previous studies have reported a full return of renal blood flow at 45 min after zone 1 cREBOA [18, 26]. Nevertheless, reducing the progressing ischemic injury to distal organs during REBOA is likely important for organ function after definite surgical care. The increased urine output after iREBOA may reflect the degree of active nephrons and thus global renal function. It is possible that the decreased ischemic time improved the renal function and increased urine output already in the immediate intensive care phase. We detected decreased renal vein pCO_2_ after reperfusion in iREBOA which suggests that the renal ischemia was lower.

We detected hemodynamic effects in both iREBOA and cREBOA similarly to previously reported. The proximal blood pressure increased from 75 to 124 mm Hg after initiation of supra-celiac REBOA in humans [27]. We also detected an increase in Troponin I in cREBOA after 60 min. Cardiac myocyte ischemia may occur after 60 min cREBOA [11]. It is possible that iREBOA reduced injury in the myocardium. An immediate decrease in heart rates occurred after balloon deflation, which was possibly a consequence of normalized intra-thoracic cardiovascular anatomy. Correspondingly, beta-blockers reduce myocardial injury in REBOA [28]. The increased need for vasopressor support on release of cREBOA may also be connected to a cardiac ischemic reperfusion injury and a decrease in central blood volume. We detected a similar effect when comparing zone 3 REBOA and an Abdominal Aortic and Junctional Tourniquet [29].

To further assess renal physiology, we analyzed renal venous blood gases in comparison with systemic arterial blood. A metabolic acidosis occurred in both cREBOA and iREBOA, showing a decrease in pH and base excess, and an increase in lactate, both systemically and in renal venous blood. There was a trend of increased metabolic acidosis in the cREBOA, although not statistically significant. Intracellular adaptation to hypoxia and ischemic preconditioning may result in decreased reperfusion injury after iREBOA which should be investigated in future studies [30]. We then analyzed immunohistochemical markers of kidney injury [31]. Osteopontin increased in the kidney cortex, and vimentin and NGAL were not affected. Osteopontin and NGAL are elevated in ischemic injury, and vimentin is elevated in AKI [32]. We specifically analyzed levels of injury markers leaked into the nephrons, by co-staining with NKCC2. Sodium–potassium-chloride cotransporters, NKCC2-pumps are located the thick ascending limb of loop of Henle [33]. It is therefore possible that the decreased urine output in cREBOA was a consequence of both decreased renal blood flow and local ischemia in the nephrons.

There are some limitations to be discussed. First, the hemorrhage protocol allowed for a standardized induction of hemorrhagic shock. However, no definitive surgical control was investigated, and no restoration of hind leg blood flow was assessed. Second, hemostasis from the transected femoral artery during reperfusion would likely not occur in humans. Extravasation through collateral pathways below the level of aortic occlusion has been described in CT scans [34], and swine without vascular pathology likely have a better vasoconstriction, in combination with a hypercoagulability, compared to humans [35]. Third, a longer follow-up during reperfusion may further characterize the return of renal blood flow and allow for a more comprehensive grading of AKI. Fourth, transferring results from animal models to humans should always be done with caution [36].

REBOA may improve mortality rates in comparison with resuscitative thoracotomy, in trauma and severe hemorrhage [37, 38]. Further improvement of resuscitation strategies and methods of REBOA to preserve renal function may improve long-term outcomes, and iREBOA shows potential as one of these methods.

## Conclusion

iREBOA was survivable, did not cause rebleeding, decreased the total ischemic time and increased the renal blood flow, urine output and decreased renal ischemic injury compared to cREBOA. Intermittent reperfusions during REBOA may be preferred to continuous, complete occlusion in prolonged application to improve renal function.

## Supplementary Information

Below is the link to the electronic supplementary material.

Supplementary file1 (DOCX 13 KB)
