# Supplementary material for: Intermittent thoracic resuscitative endovascular balloon occlusion of the aorta improves renal function compared to 60 min continuous application after porcine class III hemorrhage
Source: Eur J Trauma Emerg Surg. 2022 Dec 5;49(3):1303–13. doi: 10.1007/s00068-022-02189-2 (PMC10229682; doi:10.1007/s00068-022-02189-2)
Supplement: Supplementary file 1 — Supplementary file1 (DOCX 13 KB) [file 68_2022_2189_MOESM1_ESM.docx]

Supplemental table 1

Macro used in ImageJ for OPN/NKCC2

run("Set Measurements...", "area mean display redirect=None decimal=3");

run("Split Channels");

run("Mean...", "radius=10");

setAutoThreshold("Default dark no-reset");

//run("Threshold...");

setThreshold(400, 65535, "raw");

run("Convert to Mask");

run("Fill Holes");

run("Analyze Particles...", "size=300.00-Infinity add");

close();

run("Duplicate...", " ");

roiManager("Show None");

roiManager("Show All");

roiManager("Combine");

setBackgroundColor(0, 0, 0);

run("Clear Outside");

roiManager("Delete");

run("Mean...", "radius=5");

setAutoThreshold("Default dark no-reset");

//run("Threshold...");

setThreshold(1700, 65535, "raw");

run("Convert to Mask");

run("Analyze Particles...", "size=5-Infinity add");

roiManager("Combine");

roiManager("Delete");

roiManager("Add");

close();

roiManager("Measure");

close();

roiManager("Delete");

Macro used for NGAL/NKCC2

run("Set Measurements...", "area mean display redirect=None decimal=3");

run("Split Channels");

run("Mean...", "radius=10");

setAutoThreshold("Default dark no-reset");

//run("Threshold...");

setThreshold(550, 65535, "raw");

run("Convert to Mask");

run("Fill Holes");

run("Analyze Particles...", "size=300.00-Infinity add");

close();

run("Duplicate...", " ");

roiManager("Show None");

roiManager("Show All");

roiManager("Combine");

setBackgroundColor(0, 0, 0);

run("Clear Outside");

roiManager("Delete");

run("Mean...", "radius=5");

setAutoThreshold("Default dark no-reset");

//run("Threshold...");

setThreshold(750, 65535, "raw");

run("Convert to Mask");

run("Analyze Particles...", "size=5-Infinity add");

roiManager("Combine");

roiManager("Delete");

roiManager("Add");

close();

roiManager("Measure");

close();

roiManager("Delete");

Macro used for vimentin/NKCC2

run("Set Measurements...", "area mean display redirect=None decimal=3");

run("Split Channels");

run("Mean...", "radius=10");

setAutoThreshold("Default dark no-reset");

//run("Threshold...");

setThreshold(600, 65535, "raw");

run("Convert to Mask");

run("Fill Holes");

run("Analyze Particles...", "size=300.00-Infinity add");

close();

run("Duplicate...", " ");

roiManager("Show None");

roiManager("Show All");

roiManager("Combine");

setBackgroundColor(0, 0, 0);

run("Clear Outside");

roiManager("Delete");

run("Mean...", "radius=5");

setAutoThreshold("Default dark no-reset");

//run("Threshold...");

setThreshold(950, 65535, "raw");

run("Convert to Mask");

run("Analyze Particles...", "size=5-Infinity add");

roiManager("Combine");

roiManager("Delete");

roiManager("Add");

close();

roiManager("Measure");

close();

roiManager("Delete");
